# A case of dense pigment deposition of the posterior lens capsule

**DOI:** 10.1186/s12886-020-01728-y

**Published:** 2020-11-19

**Authors:** Igor Šivec Trampuž

**Affiliations:** Morela Okulisti in Optika, Center for Eye Refractive Surgery, Tehnološki Park 21, 1000 Ljubljana, Slovenia

**Keywords:** Pigmentary dispersion syndrome, Pigmentary glaucoma, Retinal nerve fiber layer, Intraocular pressure, Deposits, Posterior lens capsule, Optic neuropathy

## Abstract

**Background:**

Pigment dispersion syndrome (PDS) is a well-known entity which can lead to pigmentary glaucoma (PG). This case report presents a rare presentation of PG with bilateral dense pigment deposits of the posterior lens capsule.

**Case presentation:**

A 72-year-old male came for his first appointment due to an asymmetric worsening of visual acuity. The examination showed unilaterally severely increased intraocular pressure, bilateral dense pigment deposition of the posterior lens capsule, and a shallow unilateral optic disk excavation. Gonioscopy revealed moderate pigmentation of the angle and a concave configuration of the peripheral iris in both eyes. The standard slit lamp examination showed no transillumination defects of either iris. Optical coherence tomography showed retinal nerve fiber layer (RNFL) thinning in the peripapillary and macular regions. An antiglaucoma medication was prescribed with a good lowering effect.

**Conclusion:**

Pigment deposition of the posterior lens capsule, which has been rarely reported, is a possible important sign of PDS or PG.

## Background

Pigment dispersion syndrome (PDS) is a relatively rare and peculiar entity that can lead to a secondary elevation of intraocular pressure (IOP) and cause pigmentary glaucoma (PG). PDS is characterized by dispersion of pigment of the anterior segment, including Krukenberg’s spindles, iris transillumination defects, diffuse trabecular meshwork pigmentation, and a backward bowing of the iris [1–3]. It is hypothesized that the posterior bowing of the iris causes rubbing of the pigmented iris epithelium against lens structures, which in turn causes the liberation of pigment that reduces the outflow of the aqueous in the trabecular meshwork [4–7]. The majority of patients with PDS are asymptomatic; however, episodes of blurred vision, halos around light sources, and headaches have been described [2, 8]. PDS typically affects myopic patients between 20 and 40 years of age and can be seen equally distributed between men and women [2, 9, 10]. It is reported that it affects Caucasians more often than other races [1, 9, 11]. Many cases are sporadic; however, there are families with apparent autosomal dominant inheritance [12, 13].

Unlike PDS, PG is more prevalent in men [1]. The features are similar to PDS with an additional elevated IOP and glaucomatous optic neuropathy. PG tends to be a high-tension type of glaucoma with 12.5% of patients having an IOP over 39 mmHg at the time of the diagnosis [10, 14]. The probability of conversion from PDS to PG is 10% at 5 years and 15% at 15 years. A lifetime risk of PDS progressing to PG has been estimated between 35 and 50% [10, 15]. Visual field progression is common [10, 14]. Unlike many other forms of glaucoma PG has a tendency to progress to a final quiescent phase (also known as burn-out phase) with advancing age. Reduced pigment dispersion and IOP normalization have been reported with a reversal of iris transillumination defects [2, 16].

PDS has been reported to occur in 1.9% of a population undergoing glaucoma screening and it has been reported to represent 4.4% of all cases in a glaucoma department. Larger population-based studies would be necessary to enable a more exact estimation [1, 17].

## Case presentation

A 72-year old male was referred to our clinic for cataract surgery. The patient’s medical history revealed bilateral asymmetric visual acuity worsening in the past 2 years with a generalized blur and no other associated symptoms. He had no previous eye surgery or trauma. His medical and family histories were unremarkable. He had no known allergies and took no regular medication.

**Fig. 1:**
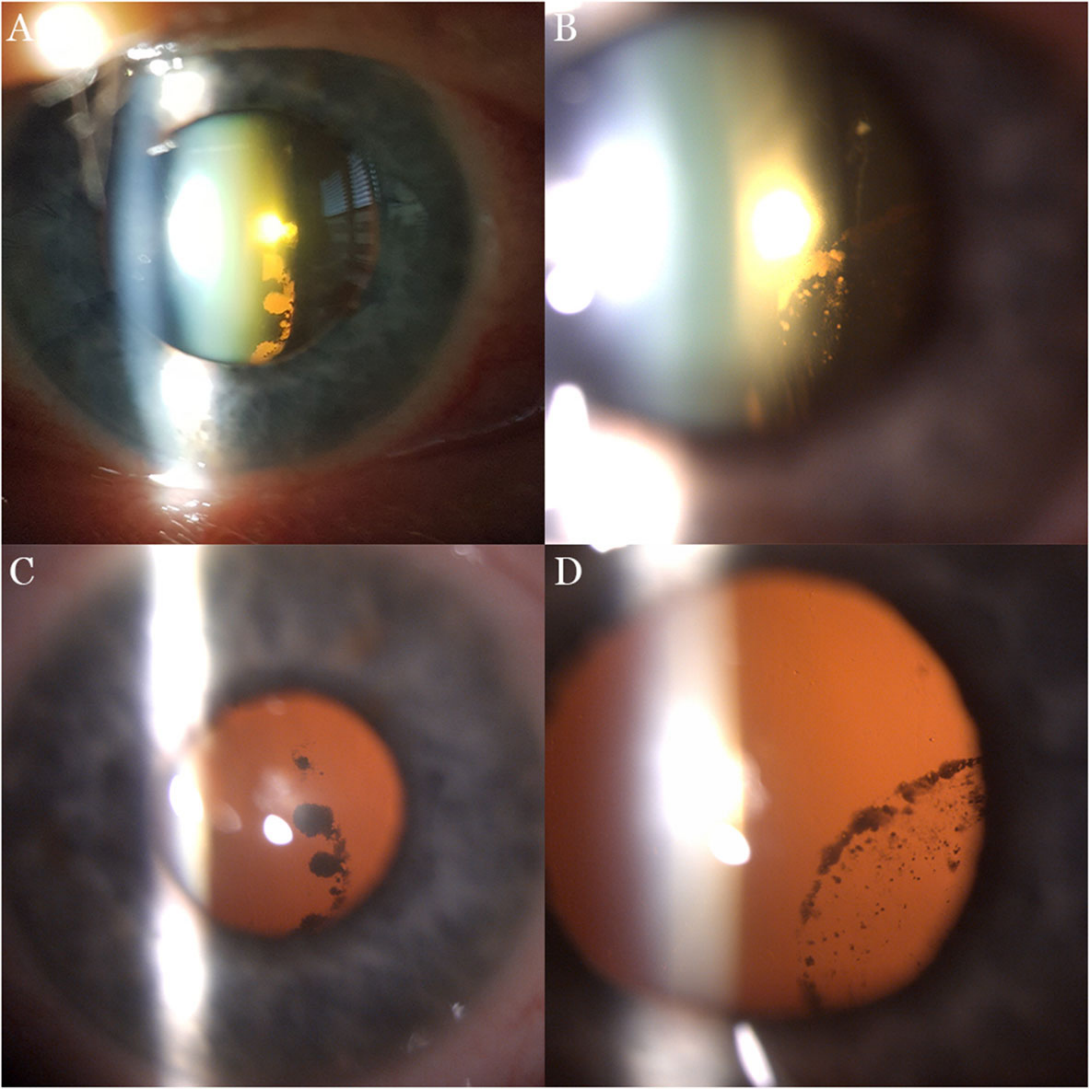
Slit lamp images. **a** Pigment deposition in right eye. **b** Pigment deposition in left eye. **c** Retroillumination of right eye. **d** Retroillumination of left eye

**Fig. 2:**
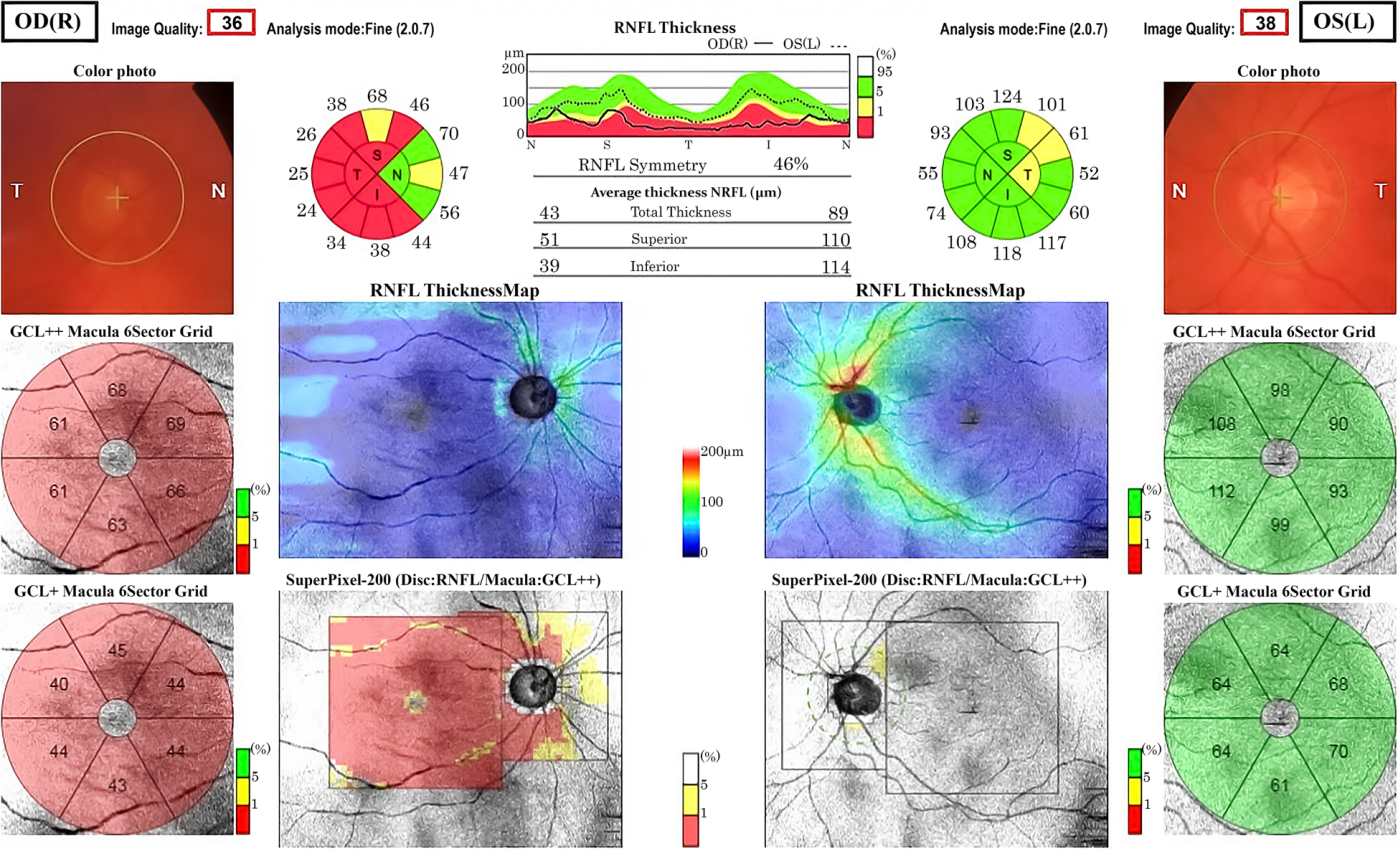
Swept source optical coherence tomography showing severe RNFL thinning in the papillary and macular region of the patients’ right eye

The ophthalmic examination showed visual acuity (VA) of 0.4 and 1.0 (Snellen chart) in his right and left eye respectively. The subjective refraction was − 3.50 - 1.00 × 80 in the right eye and − 2.25 in the left eye. Intraocular pressure measured with a Goldmann applanation tonometer was 48 mmHg in his right eye and 20 mmHg in his left eye. The patient had blue irises. The external examination was normal, but the slit lamp examination revealed dense asymmetric pigmentation of the posterior lens capsule in both eyes, although the examination of the iris was unremarkable (Fig. 1). Gonioscopy showed bilateral wide-open angles with a moderate diffuse grade 2+ pigmentation using Scheie’s grading, a pigmented line at Schwalbe’s line similar to Sampaolesi’s line, and a concavity of the peripheral iris. Dilated fundus examination showed a clear vitreous and a shallow extensive optic nerve head excavation in his right eye. He had pavingstone degenerative changes in the periphery; the rest of the fundus examination was unremarkable. The visual fields by standard static perimetry of the right eye showed generalized severe depression. Optical coherence tomography (OCT) (SS-OCT; DRI OCT Triton©Topcon, Japan) of his right eye showed generalized severe retinal nerve fiber layer (RNFL) thinning with a total thickness of 43 μm (Fig. 2). The left eye showed mild RNFL thinning with a total thickness of 89 μm. A diagnosis of pigmentary glaucoma of the right eye and pigment dispersion syndrome of the left eye was made.

Due to a severely elevated IOP we decided to prescribe the patient with a prostaglandin analogue (latanoprost once daily) for both eyes and a combination of an α_2_ adrenergic agonist and a β adrenergic antagonist (brimonidine tartrate/timolol maleate twice daily) for his right eye. The patient had a good response to topical medication. At his last follow up examination 6 months after the presentation, his VA was 0.5–0.6 and 1.0 and IOP 9 mmHg and 10 mmHg in his right and left eye respectively. OCT showed no additional RNFL thinning; also the visual fields defects were unchanged.

## Discussion and conclusion

Pigment deposition of the more central aspect of the posterior lens capsule can be the first sign of pigmentary glaucoma or pigment deposition syndrome. This clinical presentation has been rarely reported. A search of published literature using PubMed and the Cochrane Registry revealed six cases with similar clinical presentation; however, they all described cases that had unilateral pigment deposits. Roberts et al. [18], Lin et al. [19], Turgut et al. [20], and Nagarajaiah et al. [21] described cases of unilateral pigment deposition on the posterior lens capsule without ocular trauma. Al-Mezaine [22] described a case of pigment deposition after blunt ocular trauma. There is only one documented case of bilateral pigment deposition of the posterior lens capsule published by Canestraro et al. [23]. The patient presented in this latter case study had optic nerve hypoplasia which made the assessment of the damage done by the glaucoma difficult. The hypothesized explanations of the etiology of pigment deposits of the posterior lens capsule are very similar across different authors. However, due to only a small number of reported cases, it is still too early to say that we understand the pathophysiological mechanisms behind this pathological finding. An intact anterior hyaloid face exhibits a firm annular adhesion to the posterior lens capsule at Wieger’s ligament [24]. This forms a natural barrier which prevents pigment from traveling from the posterior chamber to the polar retrolental space. The pigmentation of the lens capsule is classically in the periphery in the shape of Scheie’s or Zentmayer’s line in pigment dispersion syndrome [25–27]. This means that an anatomical anomaly or a defect in Wiegers ligament would have to be present and for the aqueous from the posterior chamber to be able to flow into the retrolental space [18–20, 22]. Canestraro et al. [23] proposed that anatomical changes due to the proximity of the posterior zonulae insertions and Wieger’s ligament are to be blamed.

The identification of pigment deposition of the posterior lens capsule is important especially if other signs of PDS are subtle. This is especially true if the patient has no transillumination defects or Krukenberg’s spindle. One could easily give the patient a diagnosis of primary open angle glaucoma; however, it has a different natural course and surgical treatment modalities from PG which makes it an important entity do diagnose correctly.

## Data Availability

All data generated or analysed during this study are included in this article.

## Abbreviations

PDS: Pigment dispersion syndrome
PG: Pigmentary glaucoma
IOP: Intraocular pressure
RNFL: Retinal nerve fiber layer
OCT: Optical coherence tomography
